# Synthesis, crystal structure, and thermal properties of poly[aqua­(μ_5_-2,5-di­carb­oxy­benzene-1,4-di­carboxyl­ato)strontium]

**DOI:** 10.1107/S2056989020002005

**Published:** 2020-02-14

**Authors:** Samia Mokhtari, Chahrazed Trifa, Sofiane Bouacida, Chaouki Boudaren, Mohammed S.M. Abdelbaky, Santiago García-Granda

**Affiliations:** a Unité de Recherche de Chimie de l’Environnement et Moléculaire Structurale, CHEMS, Faculté des Sciences Exactes, Université des Frères Mentouri Constantine, 25000, Algeria; b Département Sciences de la Matière, Faculté des Sciences Exactes et Sciences de la Nature et de la Vie, Université Oum El Bouaghi 04000, Algeria; c Departamento de Química Física y Analítica, Universidad de Oviedo-CINN, 33006 Oviedo, Spain

**Keywords:** crystal structure, coordination polymer, hydro­thermal synthesis, thermal analysis

## Abstract

A coordination polymer formulated as [Sr(H_2_BTEC)(H_2_O)]_*n*_ (H_4_BTEC = benzene-1,2,4,5-tetra­carb­oxy­lic acid, C_10_H_6_O_8_), was synthesized hydro­thermally and characterized by single-crystal and powder X-ray diffraction, scanning electron microscopy and thermal analysis. Its crystal structure is made up of a zigzag inorganic chain formed by edge-sharing of [SrO_8_] polyhedra running along [001]. Adjacent chains are connected to each other *via* the carboxyl­ate groups of the ligand, resulting in a double-layered network extending parallel to (100). O—H⋯O hydrogen bonds of medium-to-weak strength between the layers consolidate the three-dimensional structure. One of the carb­oxy­lic OH functions was found to be disordered over two sets of sites with half-occupancy.

## Abstract 2

In the title polymer, the eightfold-coordinated Sr^II^ atoms are linked by bridging (H_2_BTEC)^2–^ anions to generate a two-dimensional network Thermal analysis (TG–SDTA–MS) has been undertaken.

## Chemical context   

In recent years, the self-assembly of coordination polymers (CP) and crystal engineering of metal–organic coordination frameworks have attracted great inter­est because of their varied mol­ecular topologies and the potential applications of these polymers as functional materials (Pan *et al.*, 2004 ▸; Jiang *et al.*, 2011 ▸; Du *et al.*, 2014 ▸). Derivatives of aromatic tetra­carb­oxy­lic acids such as 1,2,4,5-benzene­tetra­carb­oxy­lic acid (H_4_BTEC, commonly known as pyromellitic acid) and their deprotonated forms (H_*n*_BTEC^(4–*n*)–^) belong to an important family of polycarboxyl­ate O-donor ligands, which have been used extensively to prepare CPs (Liu *et al.*, 2009 ▸). The variations in the possible binding modes of its four potentially coordinating carb­oxy­lic/carboxyl­ate groups, along with the different coordination preferences of the metal ions, gives rise to a great variety of crystal structures.
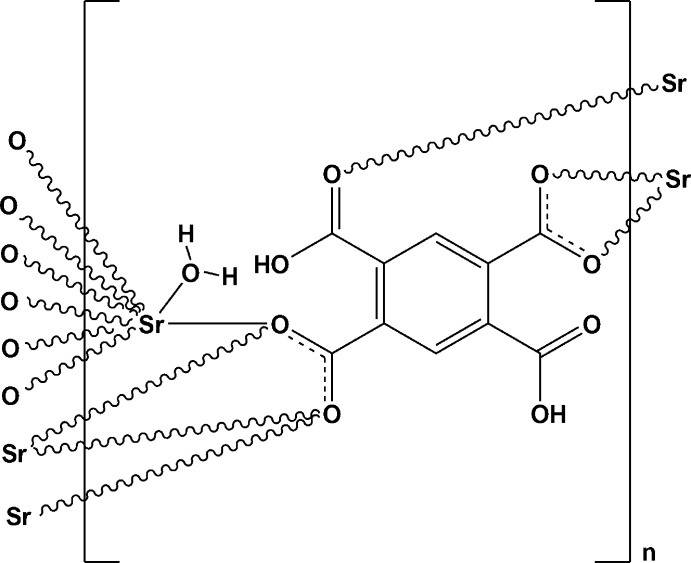


In this communication, we report on the synthesis of [Sr(H_2_BTEC)(H_2_O)], (I), along with its characterization by single-crystal and powder X-ray diffraction, scanning electron microscopy coupled with energy-dispersive X-ray fluorescence, and thermal analysis.

## Structural commentary   

**Figure 1:**
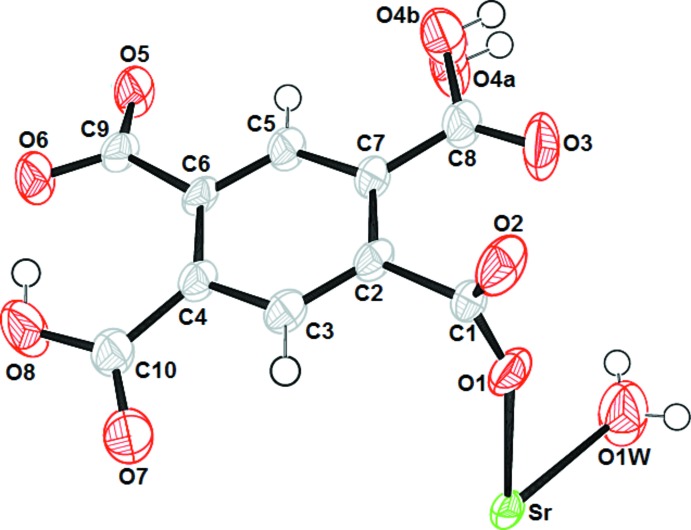
The asymmetric unit of (I), showing the atom-numbering scheme. Displacement ellipsoids are drawn at the 50% probability level. (Hy­droxy atom O4 is disordered with a 0.5:0.5 ratio.)

**Figure 2:**
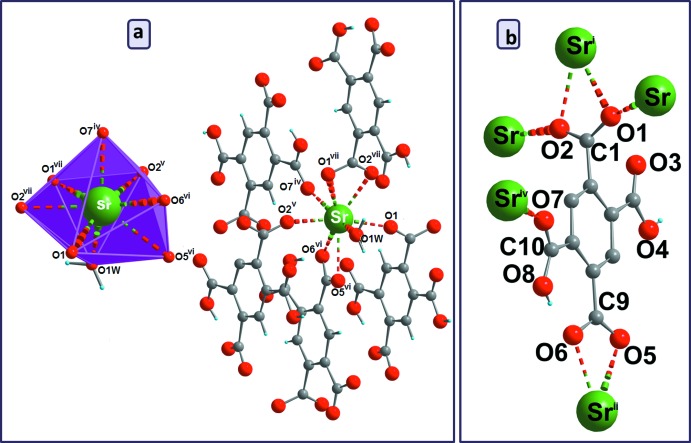
(*a*) Perspective view of the coordination environment of Sr^II^ and (*b*) coordination modes of the (H_2_BTEC)^2–^ anion in (I). [Symmetry codes: (i) *x*, −*y* + 2, *z* − 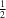; (ii) *x*, −*y* + 1, *z* − 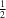; (iv) −*x* + 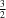, −*y* + 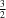, −*z* + 2; (v) *x*, *y*, *z* + 1; (vi) *x*, −*y* + 1, *z* + 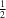; (vii) *x*, −*y* + 2, *z* + 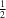.]

**Figure 3:**
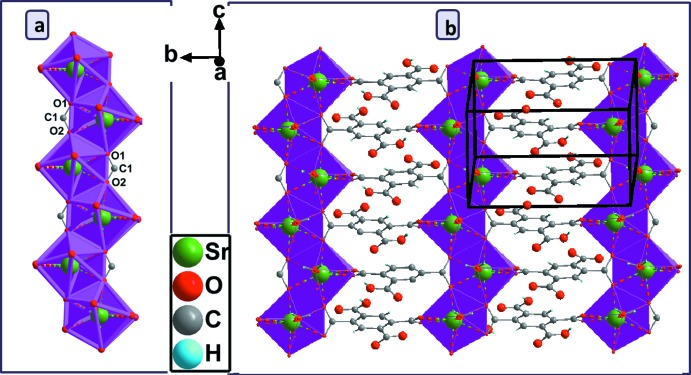
(*a*) View of the inorganic chain and (*b*) the two-dimensional layer structure in the crystal structure of (I).

**Figure 4:**
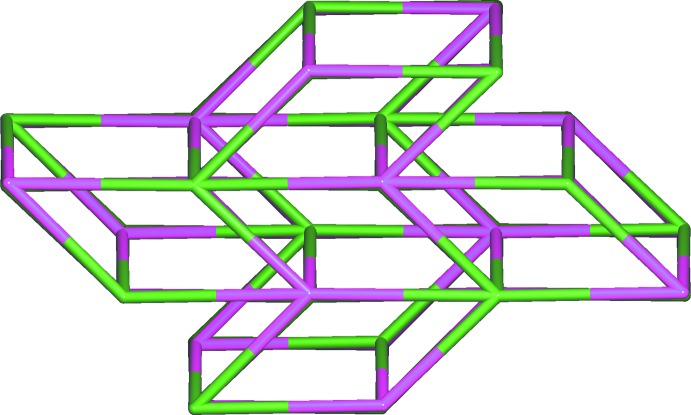
The uninodal five-connected net for (I).

The asymmetric unit of compound (I) comprises one Sr^II^ atom, one doubly deprotonated (H_2_BTEC)^2–^ anion and one coordinating water mol­ecule O1*W* (Fig. 1 ▸). The Sr^II^ atom is bonded to eight oxygen atoms, seven of them coming from five carboxyl­ate or carb­oxy­lic groups of five different (H_2_BTEC)^2–^ ligands, and one oxygen atom from the water mol­ecule. The resulting coordination polyhedron around the alkaline earth cation may be described as a distorted bicapped prism (Fig. 2 ▸ *a*). The Sr—O bond lengths span the range 2.4915 (19)–2.8239 (19) Å for carboxyl­ate/carb­oxy­lic acid groups, and the Sr—O_(water)_ bond length is 2.520 (3) Å. These distances are comparable to those reported in other strontium–carboxyl­ate complexes (He *et al.*, 2014 ▸). The (H_2_BTEC)^2–^ anion has a bridging character and connects five Sr^II^ atoms (Fig. 2 ▸ *b*) whereby three different coordination modes are realized. The carboxyl­ate group (O1—C1—O2) adopts both a bis-monodentate bridging mode to two Sr^II^ atoms and a bidentate chelating mode to a third Sr^II^ atom; the carb­oxy­lic group (O7/C10/O8/H8) is monodentately bound through O7 to a fourth Sr^II^ atom and shows an intra­molecular O8—H8⋯O6 hydrogen bond (Table 1 ▸); the carboxyl­ate group (O5/C9/O6) exhibits a bidentate chelating mode to a fifth Sr^II^ atom. The carb­oxy­lic group (O3/C8/O4/H4) has a disordered hydroxyl group and does not bind to a cation. The [SrO_8_] polyhedra share edges through (O1—O2), thus forming an infinite zigzag chain running parallel to [001] (Fig. 3 ▸ *a*). These chains are further connected through the carboxyl­ate groups (O1/C1/O2 and O5/C9/O6) into double layers parallel to (100) that are stacked along [100] (Fig. 3 ▸ *b*). A topological analysis (Blatov *et al.*, 2014 ▸) revealed that the overall structure of the coordination polymer (I) can be defined as a uninodal five-connected net with the Schläfli symbol {4^8^.6^2^}, and the vertex symbols of Sr^II^ and (H_2_BTEC)^2–^ node is [4.4.4.4.4.4.4.4.6(3).6(3)] (Fig. 4 ▸).

## Supra­molecular features   

**Figure 5:**
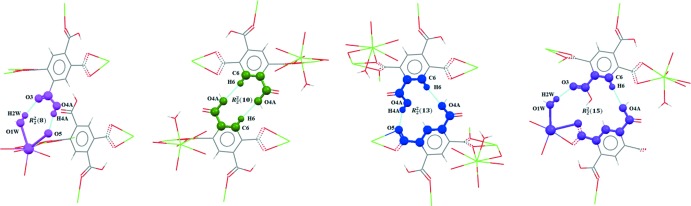
The hydrogen-bonded-ring patterns found in (I).

**Figure 6:**
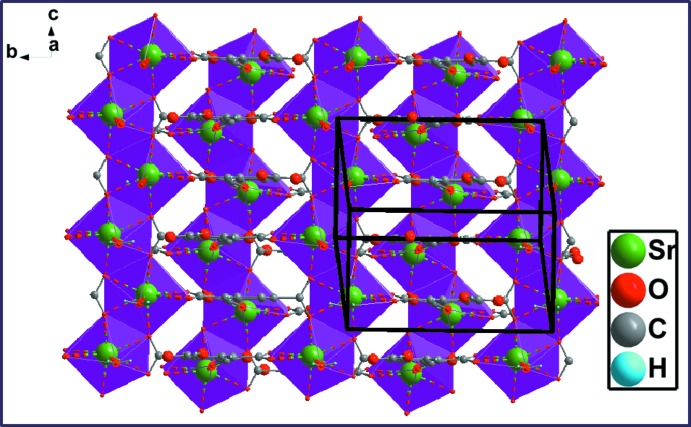
View of the double-layered network along the *a* axis.

In the crystal structure of (I), neighbouring layers are linked to each other along the stacking direction by inter­molecular O—H⋯O hydrogen bonds of medium-to-weak strength involving the coordinating water mol­ecule with the carbonyl O atom (O3) of the non-coordinating carb­oxy­lic acid group as acceptor, as well as the disordered O4—H4 function of this carb­oxy­lic acid group and carboxyl­ate O atom O4 as an acceptor group (Table 1 ▸). The hydrogen-bonding scheme is completed by two weak inter­molecular C—H⋯O inter­actions involving aromatic H atoms (Table 1 ▸). Based on the connectivity of these hydrogen bonds, four different motifs (Etter *et al.*, 1990 ▸) can be distinguished, *viz.* 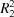
(8), 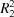
(10), 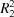
(13) and 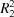
(15) (Fig. 5 ▸), leading to a three-dimensional supra­molecular structure (Figs. 6 ▸, 7 ▸).

## Crystal morophology and characterization   

**Figure 8:**
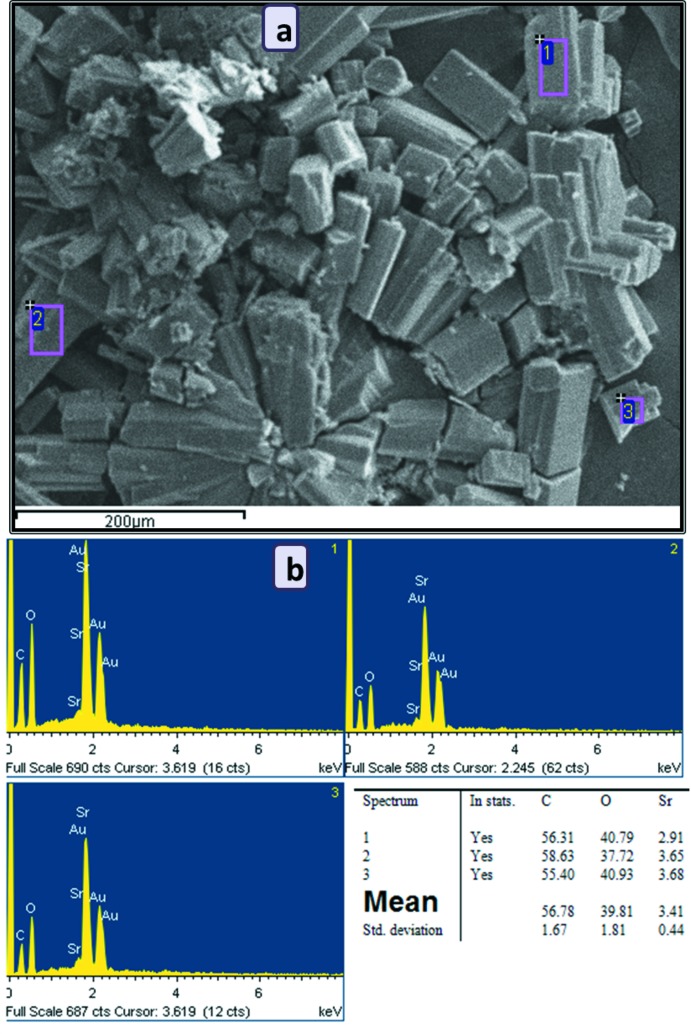
(*a*) SEM images and (*b*) a typical EDX spectrum with a table of the qu­anti­tative analysis results for Sr, O and C (in at%).

SEM images show the appearance of the microcrystalline powder, while EDX measurements provided qualitative confirmation about the presence of all non-hydrogen atoms (Fig. 8 ▸). The FT–IR spectrum of complex (I) (Fig. S1 in the supporting information) shows broad absorption bands near 3440 cm^−1^, which are assigned to O—H stretching vibrations of the –COOH groups and water mol­ecules, respectively. The bands located at 3164 cm^−1^ can be attributed to aromatic C—H stretching vibration. In addition, the symmetric [ν_s_(OCO) = 1414 and 1346 cm^−1^] and asymmetric [ν_as_(OCO) = 1626 and 1533 cm^−1^] stretching vibrations in (I) can be attributed to the split of the absorption bands of the carboxyl­ate groups. The Δ(ν_as_–ν_s_) values of 187–212 cm^−1^ indicate that some of the carboxyl­ate groups are monodentate and bridging to the Sr^II^ atoms. A strong absorption at 1731 cm^−1^ confirms the presence of the carb­oxy­lic acid function. All these results are in agreement with the crystallographic data.

**Figure 9:**
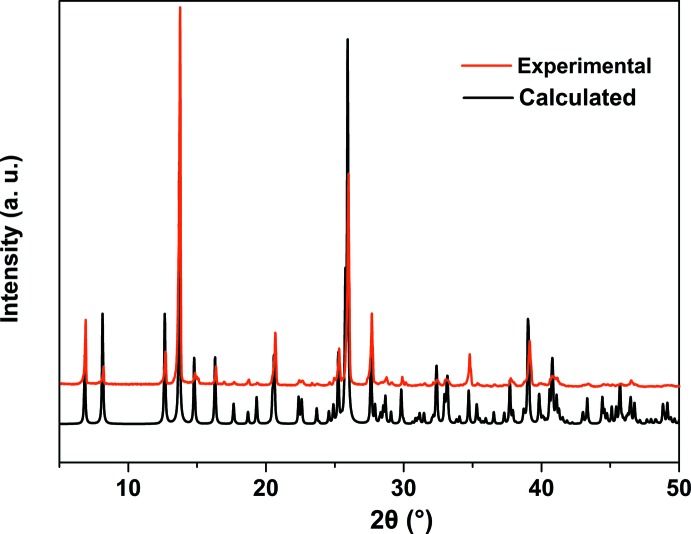
Powder XRD patterns of (I) compared with the calculated one.

**Figure 10:**
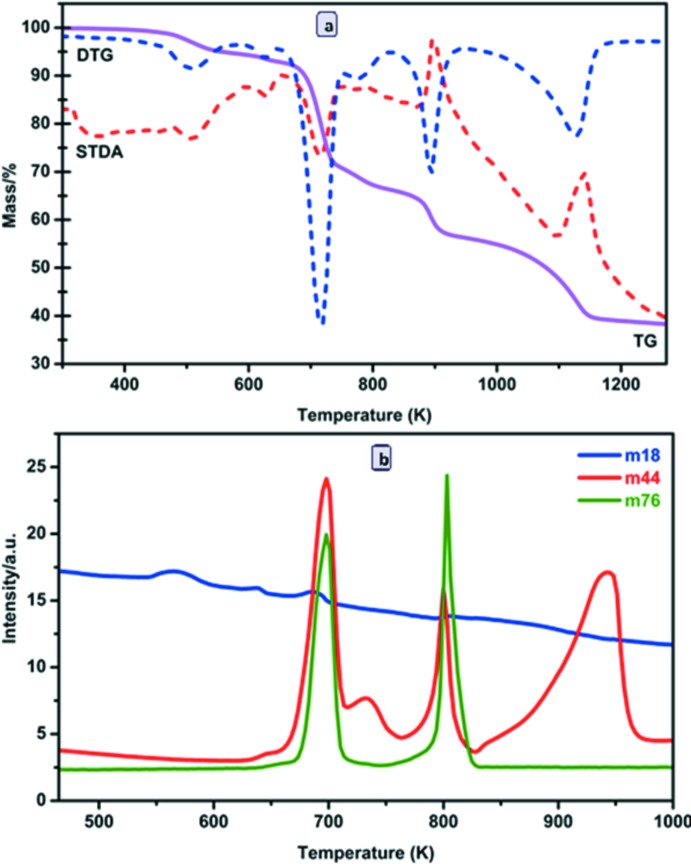
(*a*) TG–DTG–SDTA curves and (*b*) *m*/*z* 18 (H_2_O), *m*/*z* 44 (CO_2_) and *m*/*z* 76 (C_6_H_4_) MS signals for (I).

Plots of the experimental and simulated powder X-ray diffraction (PXRD) patterns of the title compound are shown in Fig. 9 ▸, revealing a good match and thus phase purity and repeatable synthesis. TG/DTG, SDTA curves and the mass spectrometry analysis are depicted in Fig. 10 ▸ *a*. TG/DTG curves of (I) reveal a total mass loss of *ca* 60.5% (calc. 58.1%) from room temperature up to 1273 K, with SrO as the final product. The mass loss of (I), under a dry N_2_ atmosphere, proceeds in four steps. The first one, between 298 and 550 K with a mass loss of *ca* 5.2% (cal. 5.0%), is associated with an endothermic reaction (491 K in the SDTA curve) and corres­ponds to the loss of the coordinating water mol­ecule. The second step, between 557 and 719 K with a mass loss of *ca* 22.1% (calc. 25.7%) and an endothermic reaction (peak at 609 K), is attributed to the beginning of the decomposition of the (H_2_BTEC)^2–^ ligand. The third step, between 706 and 908 K with a mass loss of about 15.3% is exothermic (peak at 882 K), and may be attributed to the complete decomposition of the organic anion. The fourth step, between 908 and 1147 K with a mass loss of 17.9% is also exothermic (peak at 1121 K), and may be due to another evaporation of trapped organic moieties. The associated mass spectroscopy *m*/*z* 18 (H_2_O), 44 (CO_2_), and 76 (C_6_H_4_) curves (Fig. 10 ▸ *b*) are in agreement with the TG/DTG data. The *m*/*z* 18 curve has four maxima, the first and second maxima at 565 and 639 K correspond to the loss of the coordinating water mol­ecules. The third maximum at 682 K coincides with the *m*/*z* 44 and 76 curves, which is attributed to the first decomposition step of the organic anion, and the last maximum at 806 K coincides with the second maximum of *m*/*z* 44 and 76.

## Database survey   

A search of the Cambridge Structural Database (CSD, version 5.40, update November 2018; Groom *et al.*, 2016 ▸) resulted in 196 hits for the (H_4_BTEC)^2–^ dianion. To the best of our knowledge, there are only two alkaline earth coordination polymers made up from the (H_2_BTEC)^2–^ dianion, *viz*. Ba(H_2_BTEC)(H_2_O)_5_]_*n*_ (Dale *et al.*, 2003 ▸) and [Sr_2_(H_2_BTEC)_2_(H_2_O)_2_]_*n*_ (Balegroune *et al.*, 2011 ▸). In the Ba compound, the alkaline earth cation displays a monocapped square-anti­prismatic coordination environment, and the coordination mode of the (H_2_BTEC)^2–^ ligand is monodentate to four cations at a time. The Sr compound is based on [SrO_8_] and [SrO_9_] polyhedra sharing edges, with the two independent (H_2_BTEC)^2–^ ligands coordinating to five- and six-metal cations, respectively. Compound (I) with its layered structure has a different set-up and is not comparable with these two previously reported structures.

## Synthesis and crystallization   

6.1. Synthesis

Chemicals were purchased from commercial sources and used without any further purification. Compound (I) was synthesized under hydro­thermal conditions. 0.26 g (1 mmol) of SrCl_2_,6H_2_O, 0.25 g (1 mmol) of pyromellitic acid (H_4_BTEC) and 0.04 g (1 mmol) of NaOH were dissolved in water (13 ml). The reaction mixture was stirred at room temperature to homogeneity and then placed in a Teflon-lined stainless vessel (40 ml) and heated to 433 K for 3 d under autogenous pressure, and afterwards cooled to room temperature. The resulting product of plate-like single crystals and microcrystalline powder was filtered off, washed thoroughly with distilled water, and finally air-dried at room temperature.

6.2. Experimental details

Powder X-ray diffraction patterns were recorded on a Philips X’pert diffractometer with Cu *K*α radiation. The samples were gently ground in an agate mortar in order to minimize the preferred orientation. All data were collected at room temperature over the 2θ angular range of 4–60° with a step of 0.01° and a counting time of 1.5 s per step. IR spectra were recorded with a JASCO FTIR-6300 spectrometer in the region 4000–600 cm^−1^. SEM micrographs and X-ray microanalysis (SEM/EDX) were recorded by using a JEOL-6610LV scanning electron microscope operating at 30 kV coupled with an Oxford X-Max microanalysis system (EDX). A Mettler–Toledo TGA/SDTA851e was used for the thermal analysis in a nitro­gen dynamic atmosphere (50 ml min^−1^) at a heating rate of 10 K min^−1^. In this case, *ca* 10 mg of a powder sample were thermally treated, and blank runs were performed with the empty crucible.

## Refinement   

Crystal data, data collection and structure refinement details are summarized in Table 2 ▸. C-bound hydrogen atoms were placed in idealized positions and refined with C—H = 0.93 Å and *U*_iso_ = 1.2*U*_eq_(C). The hydrogen atoms of the water mol­ecule and of the carb­oxy­lic groups were located in a difference-Fourier map and were refined with O—H = 0.93 and 0.92 Å, respectively, and with *U*_iso_(H) = 1.5*U*_eq_(O). One of the carb­oxy­lic OH functions (O4—H4) was found to be disordered over two sets of sites of equal occupancy.

## Supplementary Material

Crystal structure: contains datablock(s) SM112, I. DOI: 10.1107/S2056989020002005/wm5542sup1.cif

Structure factors: contains datablock(s) I. DOI: 10.1107/S2056989020002005/wm5542Isup2.hkl

Figure S1. DOI: 10.1107/S2056989020002005/wm5542sup3.tif

CCDC reference: 1890785

Additional supporting information:  crystallographic information; 3D view; checkCIF report

## Figures and Tables

**Figure 7:**
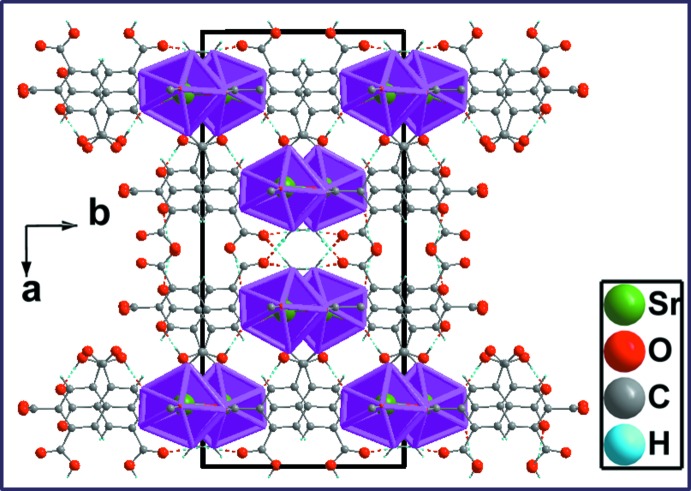
Projection of the three-dimensional structure along [001] axis with hydrogen-bonding inter­actions shown as dashed lines.

**Table 1:** Hydrogen-bond geometry (Å, °)

*D*—H⋯*A*	*D*—H	H⋯*A*	*D*⋯*A*	*D*—H⋯*A*
O1*W*—H1*W*⋯O3^i^	0.83	2.25	3.0666 (3)	170
O1*W*—H2*W*⋯O3^ii^	0.83	2.04	2.864 (4)	171
O4*A*—H4*A*⋯O5^iii^	0.82	1.92	2.68 (2)	152
O4*B*—H4*B*⋯O5^iii^	0.82	1.89	2.696 (16)	166
O8—H8⋯O6	0.82	1.59	2.400 (3)	169
C6—H6⋯O4*A* ^iii^	0.93	2.32	3.240 (18)	169
C6—H6⋯O4*B* ^iii^	0.93	2.39	3.298 (14)	166

Symmetry codes: (i) 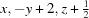; (ii) 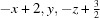; (iii) 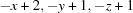.

**Table 2:** Experimental details

Crystal data	
Chemical formula	[Sr(C_10_H_4_O_8_)(H_2_O)]
*M*_r_	357.77
Crystal system, space group	Monoclinic, *C*2/*c*
Temperature (K)	295
*a*, *b*, *c* (Å)	25.8191 (7), 11.9726 (3), 7.1467 (2)
β (°)	90.662 (2)
*V* (Å^3^)	2209.05 (10)
*Z*	8
Radiation type	Mo *K*α
μ (mm^−1^)	4.93
Crystal size (mm)	0.23 × 0.14 × 0.10

Data collection	
Diffractometer	Oxford Diffraction Xcalibur, Ruby, Gemini
Absorption correction	Multi-scan (*CrysAlis PRO*; Oxford Diffraction, 2015 ▸)
*T*_min_, *T*_max_	0.833, 1.000
No. of measured, independent and observed [*I* > 2σ(*I*)] reflections	16106, 3417, 2700
*R*_int_	0.045
(sin θ/λ)_max_ (Å^−1^)	0.734

Refinement	
*R*[*F* ^2^ > 2σ(*F* ^2^)], *wR*(*F* ^2^), *S*	0.043, 0.086, 1.08
No. of reflections	3417
No. of parameters	199
No. of restraints	2
H-atom treatment	H atoms treated by a mixture of independent and constrained refinement
Δρ_max_, Δρ_min_ (e Å^−3^)	0.70, −0.41

Computer programs: *CrysAlis CCD* (Oxford Diffraction, 2015 ▸), *CrysAlis RED* (Oxford Diffraction, 2015 ▸), *SHELXS97* (Sheldrick, 2008 ▸), *SHELXL97* (Sheldrick, 2008 ▸), *ORTEP-3 for Windows* and *WinGX* (Farrugia, 2012 ▸) and *DIAMOND* (Brandenburg & Berndt, 2001 ▸).

## supplementary crystallographic information

### Crystal data

**Table:**

[Sr(C_10_H_4_O_8_)(H_2_O)]	*F*(000) = 1408
*M**_r_* = 357.77	*D*_x_ = 2.151 Mg m^−^^3^
Monoclinic, *C*2/*c*	Mo *K*α radiation, λ = 0.71073 Å
Hall symbol: -C 2yc	Cell parameters from 5087 reflections
*a* = 25.8191 (7) Å	θ = 2.9–30.9°
*b* = 11.9726 (3) Å	µ = 4.93 mm^−^^1^
*c* = 7.1467 (2) Å	*T* = 295 K
β = 90.662 (2)°	Prism, colorless
*V* = 2209.05 (10) Å^3^	0.23 × 0.14 × 0.10 mm
*Z* = 8	

### Data collection

**Table:**

Oxford Diffraction Xcalibur, Ruby, Gemini diffractometer	3417 independent reflections
Radiation source: fine-focus sealed X-ray tube, Enhance (Mo) X-ray Source	2700 reflections with *I* > 2σ(*I*)
Graphite monochromator	*R*_int_ = 0.045
Detector resolution: 10.2673 pixels mm^-1^	θ_max_ = 31.5°, θ_min_ = 2.9°
CCD rotation images, thick slices scans	*h* = −36→37
Absorption correction: multi-scan (*CrysAlis Pro*; Oxford Diffraction, 2015)	*k* = −17→16
*T*_min_ = 0.833, *T*_max_ = 1.000	*l* = −9→10
16106 measured reflections	

### Refinement

**Table:**

Refinement on *F*^2^	0 constraints
Least-squares matrix: full	Hydrogen site location: mixed
*R*[*F*^2^ > 2σ(*F*^2^)] = 0.043	H atoms treated by a mixture of independent and constrained refinement
*wR*(*F*^2^) = 0.086	*w* = 1/[σ^2^(*F*_o_^2^) + (0.0329*P*)^2^ + 3.281*P*] where *P* = (*F*_o_^2^ + 2*F*_c_^2^)/3
*S* = 1.08	(Δ/σ)_max_ = 0.007
3417 reflections	Δρ_max_ = 0.70 e Å^−^^3^
199 parameters	Δρ_min_ = −0.41 e Å^−^^3^
2 restraints	

### Special details

**Table:**

Geometry. All esds (except the esd in the dihedral angle between two l.s. planes) are estimated using the full covariance matrix. The cell esds are taken into account individually in the estimation of esds in distances, angles and torsion angles; correlations between esds in cell parameters are only used when they are defined by crystal symmetry. An approximate (isotropic) treatment of cell esds is used for estimating esds involving l.s. planes.

### Fractional atomic coordinates and isotropic or equivalent isotropic displacement parameters (Å^2^)

**Table:**

	*x*	*y*	*z*	*U*_iso_*/*U*_eq_	Occ. (<1)
C1	0.86646 (10)	0.8405 (2)	0.6041 (4)	0.0220 (5)	
C2	0.86705 (10)	0.7142 (2)	0.6075 (3)	0.0212 (5)	
C3	0.82120 (11)	0.6600 (2)	0.6499 (4)	0.0245 (6)	
H3	0.7921	0.703	0.6754	0.029*	
C4	0.81681 (10)	0.5436 (2)	0.6561 (4)	0.0222 (5)	
C5	0.86102 (10)	0.4787 (2)	0.6146 (3)	0.0206 (5)	
C6	0.90708 (11)	0.5344 (2)	0.5760 (4)	0.0252 (6)	
H6	0.9366	0.4923	0.5527	0.03*	
C7	0.91076 (11)	0.6508 (2)	0.5708 (4)	0.0240 (6)	
C8	0.96109 (11)	0.7054 (2)	0.5286 (4)	0.0310 (6)	
C9	0.86422 (11)	0.3525 (2)	0.6049 (4)	0.0253 (6)	
C10	0.76333 (11)	0.5051 (2)	0.7169 (4)	0.0290 (6)	
O1	0.86950 (8)	0.89053 (15)	0.7574 (3)	0.0306 (5)	
O2	0.86043 (9)	0.88916 (15)	0.4518 (3)	0.0346 (5)	
O1W	0.95292 (11)	0.9470 (2)	1.1096 (5)	0.0641 (8)	
H2W	0.9779 (14)	0.908 (4)	1.077 (7)	0.096*	
H1W	0.959 (2)	1.012 (2)	1.076 (7)	0.096*	
O3	0.96836 (9)	0.80320 (18)	0.5342 (5)	0.0607 (8)	
O4A	1.0004 (6)	0.6356 (8)	0.533 (7)	0.049 (6)	0.50 (7)
H4A	1.0275	0.6705	0.5217	0.073*	0.50 (7)
O4B	0.9931 (6)	0.6371 (9)	0.438 (6)	0.044 (4)	0.50 (7)
H4B	1.022	0.6653	0.4338	0.066*	0.50 (7)
O5	0.90652 (8)	0.30446 (16)	0.5932 (3)	0.0361 (5)	
O6	0.82258 (8)	0.29547 (16)	0.6041 (3)	0.0393 (5)	
O7	0.73445 (9)	0.57112 (19)	0.7873 (4)	0.0531 (7)	
O8	0.74913 (10)	0.40310 (19)	0.7001 (5)	0.0699 (10)	
H8	0.7727	0.3666	0.6541	0.105*	
Sr	0.85719 (2)	0.90596 (2)	1.10167 (3)	0.02469 (9)	

### Atomic displacement parameters (Å^2^)

**Table:**

	*U*^11^	*U*^22^	*U*^33^	*U*^12^	*U*^13^	*U*^23^
C1	0.0257 (14)	0.0109 (11)	0.0295 (13)	0.0010 (9)	0.0059 (11)	0.0014 (10)
C2	0.0295 (14)	0.0118 (11)	0.0222 (12)	0.0040 (10)	−0.0010 (11)	0.0013 (10)
C3	0.0274 (14)	0.0143 (12)	0.0318 (14)	0.0049 (10)	0.0014 (11)	0.0000 (11)
C4	0.0276 (14)	0.0143 (12)	0.0247 (13)	0.0012 (10)	0.0004 (11)	0.0000 (10)
C5	0.0283 (13)	0.0089 (10)	0.0247 (12)	0.0030 (10)	0.0017 (10)	−0.0009 (10)
C6	0.0272 (14)	0.0132 (12)	0.0352 (15)	0.0028 (10)	0.0025 (11)	0.0009 (11)
C7	0.0288 (14)	0.0114 (12)	0.0321 (14)	0.0007 (10)	0.0050 (11)	0.0014 (10)
C8	0.0293 (15)	0.0159 (13)	0.0479 (18)	0.0019 (11)	0.0065 (13)	0.0005 (12)
C9	0.0360 (16)	0.0126 (12)	0.0276 (13)	0.0001 (11)	0.0051 (12)	0.0004 (11)
C10	0.0270 (14)	0.0197 (13)	0.0405 (16)	0.0008 (11)	0.0028 (12)	−0.0007 (12)
O1	0.0493 (13)	0.0143 (9)	0.0283 (10)	−0.0005 (8)	0.0061 (9)	−0.0026 (8)
O2	0.0604 (15)	0.0153 (9)	0.0281 (10)	0.0013 (9)	−0.0010 (10)	0.0045 (8)
O1W	0.0398 (15)	0.0346 (14)	0.118 (3)	0.0015 (12)	0.0062 (16)	−0.0016 (17)
O3	0.0397 (13)	0.0185 (11)	0.124 (3)	−0.0074 (10)	0.0248 (14)	−0.0081 (13)
O4A	0.028 (3)	0.016 (2)	0.103 (18)	0.005 (2)	0.017 (6)	0.011 (5)
O4B	0.030 (4)	0.024 (3)	0.079 (12)	0.004 (2)	0.017 (5)	0.004 (4)
O5	0.0336 (11)	0.0132 (9)	0.0617 (14)	0.0025 (8)	0.0069 (10)	−0.0006 (9)
O6	0.0342 (11)	0.0135 (9)	0.0704 (15)	−0.0028 (8)	0.0131 (10)	−0.0022 (10)
O7	0.0299 (12)	0.0283 (12)	0.102 (2)	0.0011 (10)	0.0166 (13)	−0.0157 (13)
O8	0.0455 (15)	0.0218 (12)	0.143 (3)	−0.0086 (10)	0.0482 (17)	−0.0161 (14)
Sr	0.03497 (15)	0.01095 (12)	0.02837 (14)	−0.00125 (10)	0.01032 (10)	−0.00039 (10)

### Geometric parameters (Å, º)

**Table:**

C1—O2	1.243 (3)	C10—O7	1.201 (3)
C1—O1	1.251 (3)	C10—O8	1.280 (3)
C1—C2	1.512 (3)	O1—Sr	2.4915 (19)
C1—Sr^i^	3.045 (3)	O1—Sr^i^	2.6959 (19)
C2—C3	1.386 (4)	O2—Sr^iii^	2.510 (2)
C2—C7	1.388 (4)	O2—Sr^i^	2.6785 (19)
C3—C4	1.400 (4)	O1W—Sr	2.520 (3)
C3—H3	0.93	O1W—H2W	0.830 (19)
C4—C5	1.415 (3)	O1W—H1W	0.826 (19)
C4—C10	1.524 (4)	O4A—H4A	0.82
C5—C6	1.394 (4)	O4B—H4B	0.82
C5—C9	1.516 (4)	O5—Sr^ii^	2.8238 (19)
C6—C7	1.397 (3)	O6—Sr^ii^	2.572 (2)
C6—H6	0.93	O7—Sr^iv^	2.519 (2)
C7—C8	1.488 (4)	O8—H8	0.82
C8—O3	1.187 (3)	Sr—O2^v^	2.510 (2)
C8—O4A	1.314 (12)	Sr—O7^iv^	2.519 (2)
C8—O4B	1.337 (12)	Sr—O6^vi^	2.572 (2)
C9—O5	1.238 (3)	Sr—O2^vii^	2.6785 (19)
C9—O6	1.273 (3)	Sr—O1^vii^	2.6959 (19)
C9—Sr^ii^	3.099 (3)	Sr—O5^vi^	2.8239 (19)
			
O2—C1—O1	123.3 (2)	C1—O2—Sr^iii^	155.89 (18)
O2—C1—C2	119.0 (2)	C1—O2—Sr^i^	94.78 (16)
O1—C1—C2	117.7 (2)	Sr^iii^—O2—Sr^i^	108.93 (7)
O2—C1—Sr^i^	61.23 (14)	Sr—O1W—H2W	131 (4)
O1—C1—Sr^i^	62.05 (13)	Sr—O1W—H1W	112 (4)
C2—C1—Sr^i^	176.01 (17)	H2W—O1W—H1W	107 (5)
C3—C2—C7	118.9 (2)	C8—O4A—H4A	109.5
C3—C2—C1	117.6 (2)	C8—O4B—H4B	109.5
C7—C2—C1	123.5 (2)	C9—O5—Sr^ii^	90.82 (16)
C2—C3—C4	122.9 (2)	C9—O6—Sr^ii^	102.06 (16)
C2—C3—H3	118.6	C10—O7—Sr^iv^	142.5 (2)
C4—C3—H3	118.6	C10—O8—H8	109.5
C3—C4—C5	118.3 (2)	O1—Sr—O2^v^	167.19 (7)
C3—C4—C10	112.6 (2)	O1—Sr—O7^iv^	116.75 (9)
C5—C4—C10	129.0 (2)	O2^v^—Sr—O7^iv^	73.47 (9)
C6—C5—C4	118.1 (2)	O1—Sr—O1W	84.28 (10)
C6—C5—C9	114.9 (2)	O2^v^—Sr—O1W	88.39 (10)
C4—C5—C9	127.0 (2)	O7^iv^—Sr—O1W	153.66 (10)
C5—C6—C7	122.7 (2)	O1—Sr—O6^vi^	89.16 (7)
C5—C6—H6	118.6	O2^v^—Sr—O6^vi^	85.74 (7)
C7—C6—H6	118.6	O7^iv^—Sr—O6^vi^	76.84 (7)
C2—C7—C6	119.0 (2)	O1W—Sr—O6^vi^	121.57 (8)
C2—C7—C8	120.8 (2)	O1—Sr—O2^vii^	70.61 (6)
C6—C7—C8	120.2 (2)	O2^v^—Sr—O2^vii^	118.11 (5)
O3—C8—O4A	120.3 (7)	O7^iv^—Sr—O2^vii^	93.44 (8)
O3—C8—O4B	121.4 (6)	O1W—Sr—O2^vii^	78.18 (9)
O3—C8—C7	124.4 (3)	O6^vi^—Sr—O2^vii^	150.91 (7)
O4A—C8—C7	113.1 (5)	O1—Sr—O1^vii^	117.32 (5)
O4B—C8—C7	112.1 (5)	O2^v^—Sr—O1^vii^	70.04 (6)
O5—C9—O6	119.8 (2)	O7^iv^—Sr—O1^vii^	83.01 (7)
O5—C9—C5	121.0 (2)	O1W—Sr—O1^vii^	72.76 (8)
O6—C9—C5	119.2 (2)	O6^vi^—Sr—O1^vii^	152.13 (7)
O5—C9—Sr^ii^	65.65 (14)	O2^vii^—Sr—O1^vii^	48.19 (6)
O6—C9—Sr^ii^	54.25 (13)	O1—Sr—O5^vi^	81.37 (6)
C5—C9—Sr^ii^	173.12 (18)	O2^v^—Sr—O5^vi^	86.56 (7)
O7—C10—O8	119.3 (3)	O7^iv^—Sr—O5^vi^	121.96 (7)
O7—C10—C4	119.3 (3)	O1W—Sr—O5^vi^	74.46 (8)
O8—C10—C4	121.4 (2)	O6^vi^—Sr—O5^vi^	47.19 (6)
C1—O1—Sr	153.31 (17)	O2^vii^—Sr—O5^vi^	142.39 (6)
C1—O1—Sr^i^	93.76 (15)	O1^vii^—Sr—O5^vi^	139.83 (6)
Sr—O1—Sr^i^	108.96 (7)		
			
O2—C1—C2—C3	−96.5 (3)	C6—C7—C8—O4B	22 (2)
O1—C1—C2—C3	79.9 (3)	C6—C5—C9—O5	10.9 (4)
O2—C1—C2—C7	83.5 (3)	C4—C5—C9—O5	−169.8 (3)
O1—C1—C2—C7	−100.0 (3)	C6—C5—C9—O6	−167.3 (2)
C7—C2—C3—C4	−0.5 (4)	C4—C5—C9—O6	12.0 (4)
C1—C2—C3—C4	179.5 (2)	C3—C4—C10—O7	−14.4 (4)
C2—C3—C4—C5	−0.9 (4)	C5—C4—C10—O7	162.8 (3)
C2—C3—C4—C10	176.7 (2)	C3—C4—C10—O8	167.8 (3)
C3—C4—C5—C6	2.1 (4)	C5—C4—C10—O8	−15.0 (5)
C10—C4—C5—C6	−175.0 (3)	O2—C1—O1—Sr	149.6 (3)
C3—C4—C5—C9	−177.2 (2)	C2—C1—O1—Sr	−26.7 (5)
C10—C4—C5—C9	5.8 (5)	Sr^i^—C1—O1—Sr	148.8 (4)
C4—C5—C6—C7	−2.1 (4)	O2—C1—O1—Sr^i^	0.8 (3)
C9—C5—C6—C7	177.3 (3)	C2—C1—O1—Sr^i^	−175.5 (2)
C3—C2—C7—C6	0.6 (4)	O1—C1—O2—Sr^iii^	168.9 (3)
C1—C2—C7—C6	−179.5 (3)	C2—C1—O2—Sr^iii^	−14.9 (6)
C3—C2—C7—C8	−178.7 (3)	Sr^i^—C1—O2—Sr^iii^	169.7 (5)
C1—C2—C7—C8	1.3 (4)	O1—C1—O2—Sr^i^	−0.8 (3)
C5—C6—C7—C2	0.7 (4)	C2—C1—O2—Sr^i^	175.5 (2)
C5—C6—C7—C8	−180.0 (3)	O6—C9—O5—Sr^ii^	−3.8 (3)
C2—C7—C8—O3	4.7 (5)	C5—C9—O5—Sr^ii^	178.0 (2)
C6—C7—C8—O3	−174.6 (3)	O5—C9—O6—Sr^ii^	4.3 (3)
C2—C7—C8—O4A	168 (2)	C5—C9—O6—Sr^ii^	−177.48 (19)
C6—C7—C8—O4A	−12 (2)	O8—C10—O7—Sr^iv^	−9.8 (6)
C2—C7—C8—O4B	−159.0 (19)	C4—C10—O7—Sr^iv^	172.4 (3)

Symmetry codes: (i) *x*, −*y*+2, *z*−1/2; (ii) *x*, −*y*+1, *z*−1/2; (iii) *x*, *y*, *z*−1; (iv) −*x*+3/2, −*y*+3/2, −*z*+2; (v) *x*, *y*, *z*+1; (vi) *x*, −*y*+1, *z*+1/2; (vii) *x*, −*y*+2, *z*+1/2.

### Hydrogen-bond geometry (Å, º)

**Table:**

*D*—H···*A*	*D*—H	H···*A*	*D*···*A*	*D*—H···*A*
O1*W*—H1*W*···O3^vii^	0.83	2.25	3.0666 (3)	170
O1*W*—H2*W*···O3^viii^	0.83	2.04	2.864 (4)	171
O4*A*—H4*A*···O5^ix^	0.82	1.92	2.68 (2)	152
O4*B*—H4*B*···O5^ix^	0.82	1.89	2.696 (16)	166
O8—H8···O6	0.82	1.59	2.400 (3)	169
C6—H6···O4*A*^ix^	0.93	2.32	3.240 (18)	169
C6—H6···O4*B*^ix^	0.93	2.39	3.298 (14)	166

Symmetry codes: (vii) *x*, −*y*+2, *z*+1/2; (viii) −*x*+2, *y*, −*z*+3/2; (ix) −*x*+2, −*y*+1, −*z*+1.

## References

[bb1] Balegroune, F., Hammouche, A., Guehria-Laïdoudi, A., Dahaoui, S. & Lecomte, C. (2011). *Acta Cryst.* A**67**, C371.

[bb2] Blatov, V. A., Shevchenko, A. P. & Proserpio, D. M. (2014). *Cryst. Growth Des.* **14**, 3576–3586.

[bb3] Brandenburg, K. & Berndt, M. (2001). *DIAMOND*. Crystal Impact, Bonn, Germany.

[bb4] Dale, S. H., Elsegood, M. R. J. & Kainth, S. (2003). *Acta Cryst.* C**59**, m505–m508. 10.1107/s0108270103023175 14671343

[bb5] Du, M., Li, C.-P., Chen, M., Ge, Z.-W., Wang, X., Wang, L. & Liu, C.-S. (2014). *J. Am. Chem. Soc.* **136**, 10906–10909. 10.1021/ja506357n 25019403

[bb6] Etter, M. C., MacDonald, J. C. & Bernstein, J. (1990). *Acta Cryst.* B**46**, 256–262. 10.1107/s0108768189012929 2344397

[bb7] Farrugia, L. J. (2012). *J. Appl. Cryst.* **45**, 849–854.

[bb8] Groom, C. R., Bruno, I. J., Lightfoot, M. P. & Ward, S. C. (2016). *Acta Cryst.* B**72**, 171–179. 10.1107/S2052520616003954 PMC4822653 27048719

[bb9] He, Y. P., Tan, Y. X. & Zhang, J. (2014). *J. Mater. Chem.* **C2**, 4436–4440.

[bb10] Jiang, H.-L., Liu, B., Lan, Y.-Q., Kuratani, K., Akita, T., Shioyama, H., Zong, F. & Xu, Q. (2011). *J. Am. Chem. Soc.* **133**, 11854–11857. 10.1021/ja203184k 21751788

[bb11] Liu, H.-K., Tsao, T., Zhang, Y.-T. & Lin, C. H. (2009). *CrystEngComm*, **11**, 1462–1468.

[bb12] Oxford Diffraction (2015). *CrysAlis PRO*, *CrysAlis CCD* and *CrysAlis RED*. Oxford Diffraction Ltd, Yarnton, England.

[bb13] Pan, L., Sander, M.-B., Huang, X.-Y., Li, J., Smith, M., Bittner, E., Bockrath, B. & Johnson, J.-K. (2004). *J. Am. Chem. Soc.* **126**, 1308–1309. 10.1021/ja0392871 14759166

[bb14] Sheldrick, G. M. (2008). *Acta Cryst.* A**64**, 112–122. 10.1107/S0108767307043930 18156677

