# Supplementary figures and images for: Synthesis, crystal structure, and thermal properties of poly[aqua­(μ5-2,5-di­carb­oxy­benzene-1,4-di­carboxyl­ato)strontium]
Source: Acta Crystallogr E Crystallogr Commun. 2020 Feb 14;76(Pt 3):354–9. doi: 10.1107/S2056989020002005 (PMC7057371; doi:10.1107/S2056989020002005)

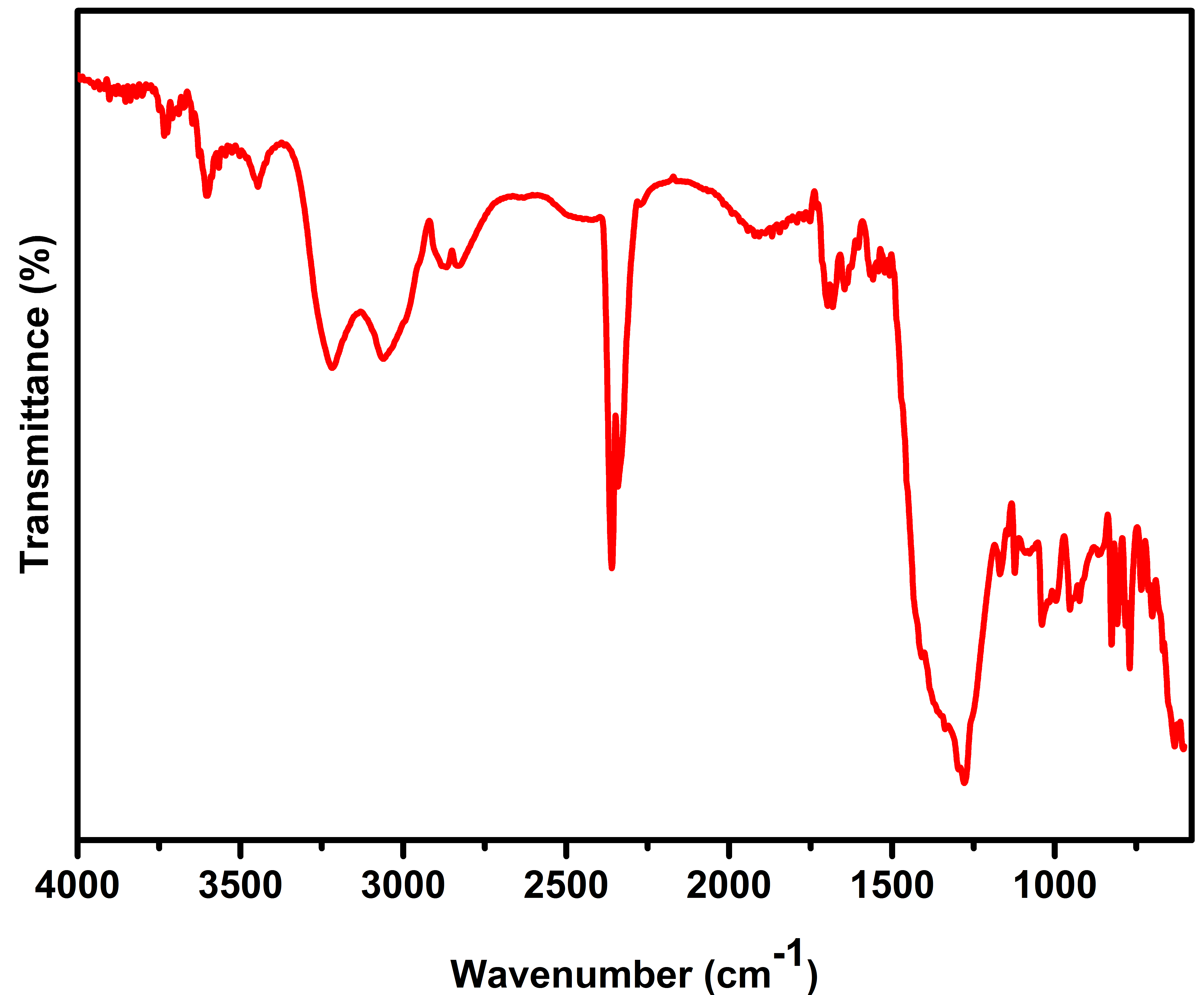

Supplement: Supplementary file 3 [file e-76-00354-sup3.tif]
